# Psychoneuroimmunology-Based Stress Management during Adjuvant Chemotherapy for Early Breast Cancer

**DOI:** 10.1155/2013/372908

**Published:** 2013-05-14

**Authors:** Jo Lynne W. Robins, Nancy L. McCain, R. K. Elswick, Jeanne M. Walter, D. Patricia Gray, Inez Tuck

**Affiliations:** ^1^Virginia Commonwealth University School of Nursing, Richmond, VA 23298, USA; ^2^Massey Cancer Center, Richmond, VA 23298, USA; ^3^North Carolina A & T University School of Nursing, Greensboro, NC 27411, USA

## Abstract

*Objective*. In a randomized trial of women with early stage breast cancer undergoing adjuvant chemotherapy, two stress management interventions, tai chi training and spiritual growth groups, were compared to a usual care control group, to evaluate psychosocial functioning, quality of life (QOL), and biological markers thought to reflect cancer- and treatment-specific mechanisms. *Method*. The sample consisted of 145 women aged 27–75 years; 75% were Caucasian and 25% African American. A total of 109 participants completed the study, yielding a 75% retention rate. Grounded in a psychoneuroimmunology framework, the overarching hypothesis was that both interventions would reduce perceived stress, enhance QOL and psychosocial functioning, normalize levels of stress-related neuroendocrine mediators, and attenuate immunosuppression. *Results*. While interesting patterns were seen across the sample and over time, the interventions had no appreciable effects when delivered during the period of chemotherapy. *Conclusions*. Findings highlight the complex nature of biobehavioral interventions in relation to treatment trajectories and potential outcomes. Psychosocial interventions like these may lack sufficient power to overcome the psychosocial or physiological stress experienced *during the chemotherapy treatment period*. It may be that interventions requiring less activity and/or group attendance would have enhanced therapeutic effects, and more active interventions need to be tested prior to and following recovery from chemotherapy.

## 1. Introduction

A growing body of research with persons having chronic and potentially fatal illnesses such as cancer indicates that a variety of complementary or “mind-body” interventions, including strategies for stress management, can not only mitigate psychological distress and improve coping skills, but also enhance immune function through neuroendocrine-immune system modulation [1]. Briefly, PNI is concerned with the mechanisms of multidimensional psychobehavioral neuroendocrine-immune system interactions. Fundamental mechanisms involve (a) the hypothalamic-pituitary-adrenocortical (HPA) system, which coordinates the release of corticotropin (ACTH), endorphins, and glucocorticoids; (b) the sympathetic nervous system via direct innervation of immune cell receptors for neurotransmitters; and (c) the sympathetic-adrenomedullary (SAM) system, which coordinates the release of catecholamines and enkephalins [2].

Serious physical illnesses such as cancer are major stressors that tend to bring about negative affective states. Glaser and Keicolt-Glaser [3] summarized convincing evidence that both psychological stress and negative emotions augment the production of proinflammatory cytokines, concluding that these stress-related changes thus have broad implications for health. Virtually all stressors, both psychological and physiological, are associated with immune activation and enhanced production of proinflammatory cytokines such as tumor-necrosis-factor-alpha (TNF-*α*), interleukin-1beta (IL-1*β*), and IL-6 [4, 5]. Other PNI-based mechanisms of increasing interest include endorphins and enkephalins, given mounting evidence that these opioid peptides have the desired response of downregulating neuroendocrine and autonomic stress responses and may counteract some aspects of cortisol-induced immunosuppression [6–8].

More women are living longer with breast cancer, which raises concerns for the quality of life of those women at diagnosis, during treatment, and beyond. The treatment period is known to be a highly stressful time for the growing population of breast cancer survivors, both psychologically and physiologically [9–11]. Women with breast cancer comprise the largest group of cancer survivors in the USA, and most are relatively young at the time of diagnosis [12], which underscores the importance of identifying strategies to enhance health and QOL in survivorship.

## 2. Methods

**Figure 1:**
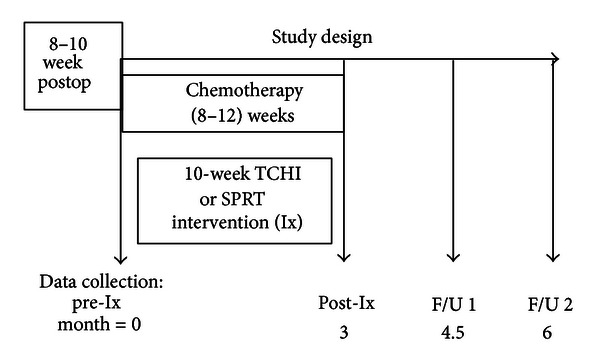
Study design.

Following informed consent and eligibility screening, participants were randomized using a computer generated randomization table, to enter the next scheduled 10-week treatment group (TCHI or SPRT) or the usual care control group. As depicted in Figure 1, data were collected at preintervention, prior to the initiation of chemotherapy (preintervention (Pre-Ix)), within a week following the intervention (postintervention (Post-Ix)), at 4.5 months (followup 1 (F/U 1)) and 6 months after enrollment (followup 2 (F/U 2)), and at comparable times for usual care control group participants.

Using a broad psychoneuroimmunology (PNI) framework [13] to examine multiple aspects of potential stress-related mechanisms, we evaluated the effects of 10-week interventions of tai chi training (TCHI) or spiritual growth groups (SPRT) in comparison to a usual care control group among women receiving adjuvant chemotherapy for stages I–IIIA breast cancer. We tested effects of these interventions on (a) enhancing psychological well-being (perceived stress, quality of life (QOL), and depressive symptoms), (b) normalizing levels of stress-related neuroendocrine mediators (endogenous opioids), and (c) attenuating immunosuppression (cytokine patterns). Previous studies using these somewhat novel interventions in persons with HIV infection documented selected psychosocial and physiological effects including enhanced QOL, increased plasma levels of interferon-gamma (IFN-*γ*), and increased lymphocyte proliferative function [14, 15]. In order to expand the application of these interventions among populations with immune-mediated or immune-moderated conditions, we sought to test the effects of these PNI-based approaches for women receiving adjuvant chemotherapy for early stage breast cancer.

### 2.1. Measures

#### 2.1.1. Psychosocial Instruments

Perceived stress related to diagnosis and treatment for breast cancer will be measured by the Impact of Events Scale (IES) [16]. Because of its specific nature and previous sensitivity to psychosocial interventions, we used the IES to measure the subjective distress of living with breast cancer. The IES has excellent psychometric properties and is not confounded with physical symptoms. It is a 15-item instrument with response options that indicate how frequently within the past 7 days each distressing thought related to having breast cancer and chemotherapy has occurred. Higher scores on the subscales of intrusive and avoidant thinking indicate greater psychological distress.

General QOL, along with QOL specific to living with breast cancer, was measured by the *Functional Assessment of Cancer Therapy-Breast* (FACT-B) cancer instrument [17]. The FACT-B (version 4) is a 44-item self-report instrument designed to assess QOL in breast cancer along the dimensions of functional well-being, physical well-being, emotional well-being, and breast cancer-specific factors. The instrument has demonstrated good validity and reliability in a variety of studies among women with breast cancer [18, 19].

Depressive symptoms are commonly measured in people with cancer by the *Center for Epidemiological Studies-Depression* (CES-D) scale [20]. The CES-D is a 20-item scale that asks participants to report the extent to which they experienced each of the symptoms in the preceding week. The CES-D has been widely used in persons with breast cancer with alpha coefficient of .88, test-retest reliability .51–.67, and convergent validity established by significant correlations with other established measures of depression [6, 21, 22].

#### 2.1.2. Neuroendocrine Measures

Endorphins and enkephalins also are stress-related neuroendocrine mediators, but little clinical or biobehavioral research examining these endogenous opioids has yet been conducted. However, there is mounting evidence that opioid peptides, which are widely distributed throughout the central, peripheral, and autonomic nervous systems as well as multiple endocrine and target tissues, downregulate neuroendocrine and autonomic stress responses [6]. Opioids have been shown to affect *in vitro *function of virtually all cells of the immune system and generally have dose-dependent effects such that low doses enhance and high doses suppress immune function. Endorphins and enkephalins also are stress-related neuroendocrine mediators, but little clinical or biobehavioral research examining these endogenous opioids has yet been conducted [24, 25]. Given the available evidence and increasing interest in positive stress responses, quantification of opioid peptides is indicated in clinical studies to begin to explore their potential roles in neuroendocrine mediation of the stress process. Participants were provided with a styrofoam ice chest in order to keep their urine specimens on ice for 12-hour overnight collection periods. Urine samples were cryopreserved and batch-assayed for levels of beta-endorphin and leu-enkephalin using commercial, standardized enzyme-linked immunosorbent assay (ELISA) kits (MD Biosciences) according to the manufacturer's protocols.

#### 2.1.3. Immunological Measures

The role of cytokines, particularly the proinflammatory cytokines involved in acute phase responses (IL-1, IL-6, and TNF-*α*), has been of considerable interest in recent research on psychobehavioral changes, commonly termed as “sickness behaviors” and including fatigue and depressed mood, in persons with cancer [26, 27]. Using Bio-Plex Pro (Bio-Rad Inc.) magnetic bead technology, levels of a standardized panel of cytokines, chemokines, and growth factors in cryopreserved plasma samples were analyzed using well-established protocols. The Bio-Rad 17-plex panel was used to measure IL-1*β*, IL-2, IL-4, IL-5, IL-6, IL-7, IL-8, IL-10, IL-12 (p70), IL-13, IL-17, granulocyte colony stimulating factor (G-CSF), granulocyte-macrophage-(GM-) CSF, IFN-*γ*, monocyte-chemotactic-protein-1 (MCP-1) (MCAF), macrophage-inflammatory-protein (MIP-) 1*β*, and tumor-necrosis-factor-alpha (TNF-*α*).

### 2.2. Interventions

Intervention groups met for 90 minutes each week for a total of 10 weeks. Participants were required to attend a minimum of 8 sessions to remain in the study. Based on prior research and in consideration of the potential physical limitations of our participants, a focused short form of tai chi training (TCHI) involving eight movements was used in this study [28]. The sequence of movements was focused on developing each individual's skills in balancing, focused breathing, gentle physical posturing and movement, and the active use of consciousness for relaxation. Movements were taught in a sequence that allowed repetitive instruction as well as a progressive building of skills. Training was designed to promote increased control of attention, increased flexibility, and an integrated mind-body relaxation experience. Additionally, the particular meanings and metaphors associated with the TCHI movements were integrated to provide a cognitive component to enhance stress management. For example, when teaching the “5 Elements,” the concepts of ongoing change and transformation are discussed as represented by the seasons of the year, and the 5 elements as wood are transformed by fire into earth, the earth gives rise to minerals, minerals become water, and water nourishes wood completing the supporting cycle of life. Training videotapes/DVDs were produced and distributed to participants for weekly and ongoing practice of the techniques. To quantify TCHI practice between sessions, weekly practice cards were distributed; however, the limited number of cards returned prevented valid assessment of the amount of practice between sessions. Additionally, while not quantifiable, participants consistently reported using and enjoying the TCHI DVDs.

The spiritual growth groups (SPRT) [29] were designed for personal exploration and group sharing of spirituality, aimed at enhancing awareness of the meaning and expression of spirituality while supporting both secular and religious views of spirituality in a group format. Each session was designed to explore an aspect of spirituality, including the intellectual process of knowing or apprehending spirituality; the experiential component of interconnecting one's spirit with self, others, nature, God, or a higher power; and an appreciation of the multisensory experience of spirituality.

### 2.3. Data Analytic Approaches

We used mixed linear modeling to compare changes in the usual care control group with each intervention group and to accommodate for the correlated structure in the repeated measures for three time periods (Pre-Ix to Post-Ix, Pre-Ix to F/U 1, and Pre-Ix to F/U 2). In an attempt to satisfy the model assumptions, the cytokines were log transformed using log⁡_e_ (natural log). No effects for potential cofactors such as age, race, menopausal status, type of therapy, and stage of disease were found in the initial modeling, and thus they were removed from the final models.

## 3. Results

The sample consisted of 145 women aged 27–75 years (average = 50  years); 75% were Caucasian and 25% African American. A total of 109 participants completed the intervention or comparable usual care control group measures, yielding a 75% retention rate. The predominant reason for attrition, involving a full 40% of withdrawals, was related to intervention group meetings (i.e., not being able or not wanting to continue group meetings). No differences were seen in the demographics for those who withdrew and those who remained in the study.

Intervention and usual care control group participants did not differ by age, race, menopausal status, diagnostic stage, or treatment approach nor by self-reported physical or functional well-being. Additionally, group participants were comparably distributed across breast cancer stage. Participants received relatively consistent protocols for chemotherapy regimens, guided by national standards and ongoing clinical trials at a National Cancer Institute-designated Cancer Center or its affiliated local institutions. Following surgical removal of their breast tumors, the majority of participants received regimens of cyclophosphamide and doxorubicin every 2-3 weeks for a total of 4 doses.

**Figure 2:**
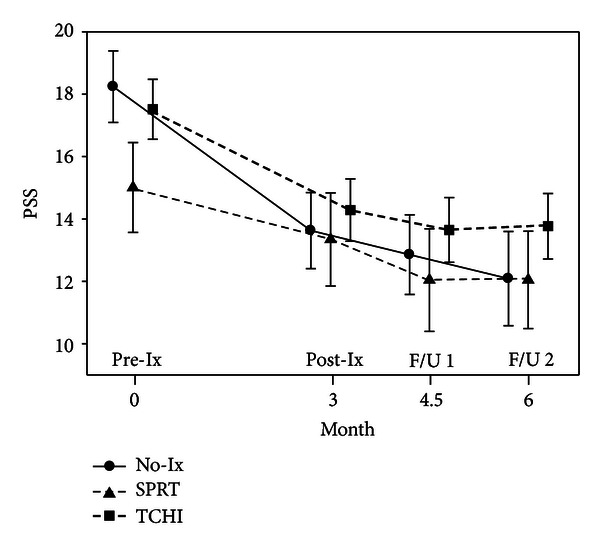
Perceived stress scores (IES) for groups over time.

**Figure 3:**
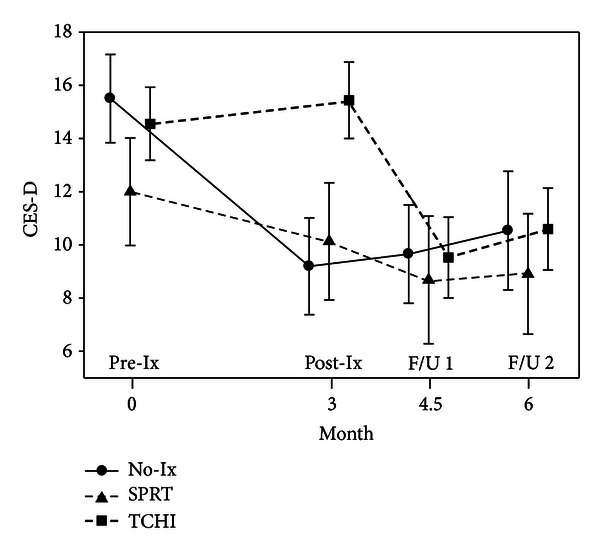
Depressive symptom scores (CES-D) for groups over time.

For all participants, levels of stress were highest at baseline, decreased over the period of chemotherapy, and then plateaued over the recovery period. Similarly, QOL scores decreased during chemotherapy but increased by the first follow-up time point.  Figure 2 illustrates this type of pattern with the IES scores, while Table 1 displays the total sample scores for the IES and FACT-B over time, none of which were different by group. However, immediately following postintervention, TCHI participants demonstrated an increase (*P* = 0.003) in depressive symptoms (mean = 15.4,  SE = 1.4) as compared to the standard care (mean = 9.2,  SE = 1.8) and SPRT (mean = 10.1,  SE = 2.2) groups. This pattern was not evident 6 weeks later at F/U 1, and levels of depressive symptoms declined for all groups of participants over time (Figure 3).

**Figure 4:**
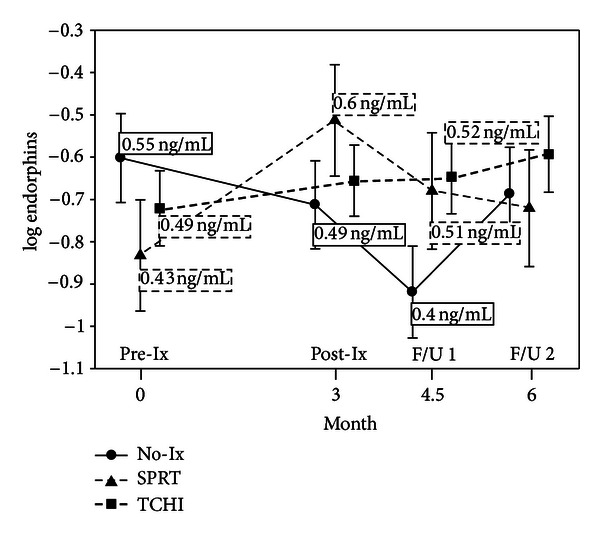
Urinary endorphin levels by group over time. Means in boxes are back-transformed from log⁡_e_ (natural log) used for analysis and are shown for timepoints with significantly different values only.

Significant elevations were seen in urinary beta-endorphin levels for the SPRT group immediately following intervention and for both SPRT and TCHI at the 4.5-month followup (Figure 4). There were no significant differences in enkephalin levels between groups at any measured time point.

**Figure 5:**
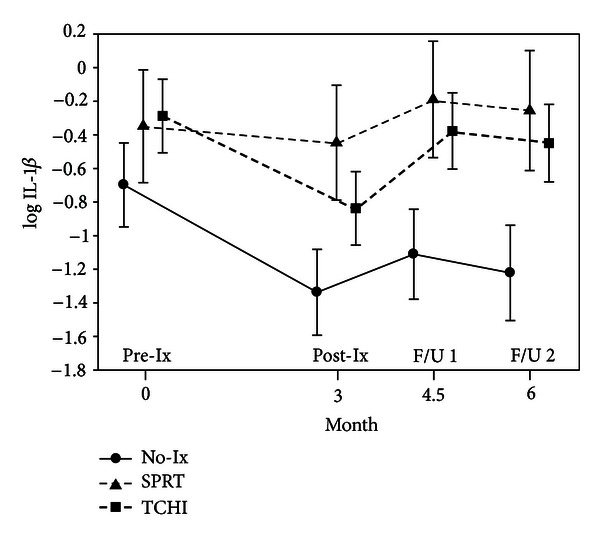
IL-1*β* levels by group over time.

**Figure 6:**
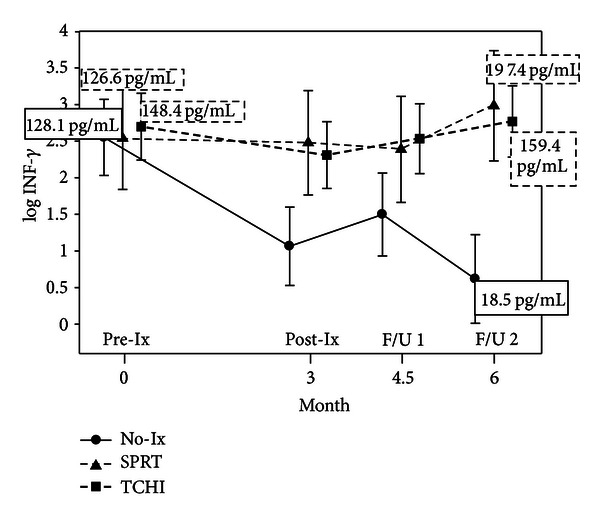
IFN-*γ* levels by group over time. Means in boxes are back-transformed from log⁡_e_ (natural log) used for analysis and are shown for timepoints with significantly different values only.

As displayed in Figure 5 for IL-1*β*, trends of declining levels during chemotherapy, followed by recovery of cytokine levels, were seen for several proinflammatory cytokines (IL-1*β*, IL-2, IL-6, and IFN-*γ*). However, the only significant differences between the two treatment groups and the usual care control group in the cytokines were seen in IFN-*γ*. As shown in Figure 6, significant elevations in IFN-*γ* were noted for both intervention groups at the 6-month followup possibly reflecting better recovery of proinflammatory cytokine production.

## 4. Discussion and Conclusions

The increase in the stress levels in TCHI participants demonstrated at postintervention when compared to the usual care control group might be an indication of increased self-awareness and/or “centering,” reflected as psychological distress. TCHI, like other mindfulness-based interventions, focuses on enhancing mindfulness and self-awareness, which may temporarily increase depressive-like symptoms in the face of critical illness situations such as cancer chemotherapy. Factors such as self-awareness and mindfulness, however, were not directly measured in this study. Additionally, TCHI groups were small, typically 3–5 women, and participants voiced discomfort related to learning a new skill in small groups. Chintamani and colleagues [10] found that response to chemotherapy was the most significant variable affecting psychological status in 84 Indian women with locally advanced breast cancer. While sample size, cultural factors, and stage of disease limit generalizability of these findings, it is reasonable to assume that response to treatment impacts psychological status in women with breast cancer. In the current study, we did not specifically track response to treatment and thus could not examine whether this was a factor contributing to study outcomes.

Limited studies of traditional stress management interventions, particularly cognitive behavioral stress management (CBSM), have been found to enhance some aspects of psychosocial and physiological function in women newly diagnosed with breast cancer. For example, Antoni and colleagues [30] found that a 10-week CBSM intervention in a sample of 128 women reduced cancer-specific anxiety and promoted physiological adaptation as evidenced by reduced cortisol and increased IL-2, IFN-*γ*, and IL-2:IL-4 ratio. Additionally, the intervention was associated with increases in participants' perceived ability to relax as well as reductions in afternoon cortisol levels during and immediately following treatment [27].

CBSM offers a variety of techniques for inducing relaxation and cognitively restructuring perceptions of and responses to stress, thereby enabling participants to choose the method that works best for them. While the TCHI intervention offered a variety of different movements, the *combination* of movements is consistent with the strength of traditional TCHI practice, and selection of individual movements is not encouraged. The SPRT intervention also offered several activities; however, each activity was focused on the exploration of one's spirituality and not necessarily congruent with inducing relaxation per se. Indeed, such interventions as TCHI and SPRT may lack the “potency” to overcome the psychological stress and physiological effects of chemotherapy itself. It may be that interventions requiring less activity and/or group attendance would have more therapeutic effects in persons receiving chemotherapy treatment regimens.

While there were significant changes in urinary beta-endorphin levels, there were no significant findings for leu-enkephalin. Given the available evidence and increasing interest in positive stress responses, quantification of endogenous opioid peptides is a promising addition in clinical studies to begin to explore their potential roles in the stress process, including immune function.

*Generally, the type, timing, and sequencing of stress management interventions have as yet undefined effects on outcomes*. Findings of this study highlight the complex nature of biobehavioral interventions in relation to treatment trajectories and potential outcomes. Psychosocial interventions like TCHI or SPRT may lack sufficient power to overcome the psychosocial or physiological stress experienced during the period of treatment with immunosuppressive chemotherapeutic agents and commonly experienced side effects such as fatigue and gastrointestinal disturbances.

Because of the inherent complexity in production, mechanisms of action, pleotropic effects, and cytokine patterns rather than levels of production of one or a selected few “representative” cytokines need to be evaluated. State-of-the-science technologies and emerging analytic approaches to evaluate patterns of cytokines are now being used to discern clinically meaningful effects and potential mechanistic insights [22, 31].

Most of the research on psychosocial interventions in breast cancer involves patients who have completed the active treatment. Ours is one of few reports of psychosocial interventions involving movement and spirituality for individuals during the period of active chemotherapy and/or radiotherapy for cancer. We undertook a “high-risk” study with respect to the delivery of somewhat novel interventions during a period of high stress and complex physical symptoms related to breast cancer and its treatment. While informal subjective feedback from participants was positive among most women who completed the interventions, the quantitative measures indicated little effect of either intervention in this study.

## Figures and Tables

**Table 1:** Psychological well-being and quality of life: means (SE) for total sample over time.

Measure	Time			
	Pre-Ix	Post-Ix	F/U 1	F/U 2
IES*	16.92 (0.69)	13.75 (0.72)	12.85 (0.78)	12.63 (0.80)
FACT-B^†^	105.19 (2.03)	102.96 (2.12)	110.19 (2.20)	110.63 (2.27)

*Means are significantly different over time (*P* < 0.0001) with Pre-Ix mean significantly greater than Post-Ix, F/U 1, and F/U 2 means.

^†^Means are significantly different over time (*P* < 0.0001) with Pre-Ix and Post-Ix means significantly greater than F/U 1 and F/U 2 means.
